# Concurrent Validity and Within-Session Reliability of a Wireless Surface Electromyography Device (MR EMG) Compared to a Criterion Measure During the Leg Extension Exercise

**DOI:** 10.3390/s26165165

**Published:** 2026-08-14

**Authors:** Jamie J. Ghigiarelli, Adam M. Gonzalez, Grace A. Valdez, Thomas L. Johannessen, Admar L. Calovini, James M. Hackney

**Affiliations:** Department of Allied Health and Kinesiology, Hofstra University, Hempstead, NY 11549, USA; adam.m.gonzalez@hofstra.edu (A.M.G.); grace.a.valdez@hofstra.edu (G.A.V.); tjohannessen1@pride.hofstra.edu (T.L.J.); acalovini1@pride.hofstra.edu (A.L.C.); james.hackney@hofstra.edu (J.M.H.)

**Keywords:** electromyography, validity, reliability, vastus lateralis, vastus medialis, leg extension, RMS amplitude

## Abstract

*Purpose:* The aim of this study was to assess within-session reliability and concurrent validity of a wireless surface electromyography device (MR EMG) relative to a wireless criterion system (Delsys Trigno) during dynamic leg extension at varying loading intensities. *Methods:* Thirty-one resistance-trained adults performed four repetitions at 30%, 60%, and 90% of their predicted one-repetition maximum (1RM) on the plate-loaded leg extension machine, with EMG amplitude recorded simultaneously by both systems for the vastus lateralis (VL) and vastus medialis obliquus (VMO). Root mean square (RMS) amplitudes were normalized to maximal voluntary isometric contraction and compared across devices using intraclass correlation coefficients (ICC), coefficients of variation (CV%), standard error of measurement, Pearson correlations, and Bland–Altman analyses. *Results:* Both devices demonstrated excellent within-session reliability (ICC = 0.92–0.97; CV = 7.8–11.3%). Validity differed by muscle. Across all loads, VMO agreement was good (ICC = 0.82–0.85; *r* = 0.83–0.86), while VL agreement was moderate (ICC = 0.53–0.57; *r* = 0.54–0.64). Bland–Altman analyses revealed systematic positive bias across all conditions, with proportional bias present for the VL but absent for the VMO. *Conclusions:* MR EMG demonstrated excellent within-session reliability but muscle-dependent concurrent validity, with good VMO agreement and moderate VL agreement with the criterion system.

## 1. Introduction

Surface electromyography (sEMG) is widely used to estimate skeletal muscle activation during clinical, laboratory, and sport-related tasks [1,2]. Accurate interpretation of sEMG amplitude, however, depends on appropriate signal acquisition and processing parameters, including sampling frequency, bandwidth, filtering strategy, and amplitude estimation method [3,4]. During dynamic resistance exercise, sEMG recordings are particularly susceptible to motion artifact, electrode displacement, and biomechanical modulation of the signal, which may alter root mean square (RMS) amplitude estimates and complicate comparisons across conditions [5,6]. Consequently, validation of emerging wearable sEMG systems must extend beyond static or low-movement conditions to ensure accurate amplitude estimation during high-force dynamic contractions.

Recent advances in wearable and low-cost sEMG technologies have increased the accessibility of muscle activation monitoring in clinical, educational, and field settings [7,8,9]. Several studies have demonstrated acceptable validity and reliability of these systems when compared with laboratory-grade equipment under controlled or isometric conditions [10,11,12,13,14,15]. The validity of low-cost systems during dynamic, high-force resistance exercise, however, remains less established, as movement-related artifacts, variations in muscle length, and contraction velocity may differentially influence amplitude-based metrics across devices [5,16]. Therefore, evaluation of these systems under progressively loaded dynamic conditions is necessary before their broader application in strength and conditioning or rehabilitation contexts.

The MR EMG system (Dunedin, New Zealand) is a wireless sEMG device that integrates smartphone-based data acquisition with onboard amplitude processing. Initial validation of the prototype device, later commercialized as MR EMG demonstrated strong agreement with conventional wired systems for recording masseter muscle activity, with intraclass correlation coefficients ranging from 0.94 to 1.00 during controlled jaw contractions [17]. Subsequent investigations have applied MR EMG in clinical and free-living contexts, including assessing the effect of clear aligners on masticatory muscle activity [18,19,20], but these studies did not independently re-establish the device’s validity against a criterion standard. The MR EMG system has also been employed to assess pectoralis major activation during dynamic barbell bench pressing [21], indicating growing interest in its application to resistance exercise. Apart from the recent bench press investigation, MR EMG validation has been conducted predominantly in low-force, constrained masticatory tasks. No study has systematically evaluated its concurrent validity against a laboratory-grade reference system during progressively loaded lower-limb resistance exercise. The contribution of the present study is therefore not novelty of the device, but rather ecological validity: establishing whether MR EMG’s validity, previously demonstrated only under low-force, constrained conditions, extends to a substantially different measurement context involving high-force, dynamic, multi-joint resistance exercise.

Dynamic resistance exercise and masticatory tasks present distinct challenges for sEMG amplitude estimation. Masticatory tasks are limited by low signal-to-noise ratio and less confident maximal voluntary contraction, whereas dynamic resistance exercise introduces motion artifact and electrode displacement. High-force cyclical contractions introduce motion artifact, electrode displacement, and continuous changes in muscle length and contraction velocity that may distort RMS amplitude estimates [3,5]. Validity observed under low-force conditions has been shown not to generalize across exercise type and intensity [16].

Therefore, the purpose of this study was to evaluate the within-session reliability and concurrent validity of the MR EMG system relative to a laboratory-grade sEMG system (Trigno™ Wireless System, Delsys, Inc., Natick, MA, USA) for measuring vastus lateralis (VL) and vastus medialis obliquus (VMO) muscle activation during dynamic leg extensions performed at 30%, 60%, and 90% of a one-repetition maximum (1RM). It was hypothesized that (1) MR EMG RMS amplitude would demonstrate good-to-excellent within-session reliability across repeated repetitions at each loading intensity [intraclass correlation coefficients (ICC) ≥ 0.75], and (2) MR EMG RMS amplitude would demonstrate good-to-excellent agreement, based on ICC consistency, with the Delsys Trigno across all loading intensities and muscles (ICC ≥ 0.75). These hypotheses predicted good-to-excellent agreement based on MR EMG’s previously demonstrated strong agreement with conventional wired systems during controlled masticatory tasks (ICC = 0.94–1.00) [17]. We expected this agreement to carry over to a new exercise context, though no study had tested it under high-force, dynamic loading.

## 2. Materials and Methods

### 2.1. Participants

A total of 31 participants were recruited from a local university, including 21 males (age: 21.0 ± 1.7 years; body mass: 83.4 ± 19.1 kg; height: 177.7 ± 8.9 cm) and 10 females (age: 21.5 ± 1.4 years; body mass: 60.6 ± 12.9 kg; height: 161.4 ± 5.1 cm). Participants reported an average of 4.7 ± 3.6 years of resistance training experience and a mean predicted leg extension 1RM of 135.1 ± 44.0 kg. Inclusion criteria were: 18 years or older, no self-reported history of cardiovascular or cerebrovascular conditions (including stroke or transient ischemic attack) on the Medical and Health History Questionnaire, free of musculoskeletal injuries within the preceding 6 months, and a minimum of one year of resistance training experience. Participants were excluded if they answered affirmatively to any question on the Medical and Health History Questionnaire or were unable to perform physical exercise. Written informed consent was obtained from all participants prior to enrollment. The study protocol was approved by the university Institutional Review Board (20251112-AHK-SHS-GHI-1).

An a priori sample size estimate was determined using the reference table provided by Bujang and Baharum [22], for ICC-based reliability studies, specifying a null ICC of 0.70 and an expected ICC of 0.90 [23], with α = 0.05, power = 0.80, and two measurement occasions. A minimum of 19 participants was required; the enrolled sample of 31 exceeded this requirement. The primary concurrent validity comparison was defined a priori as the ICC between devices for the VMO RMS amplitude at 60% 1RM. This comparison was selected a priori because the VMO offers a more consistent electrode site than the VL, and 60% 1RM represents a moderate, practically representative training load for the leg extension exercise.

### 2.2. Experimental Procedures

This study assessed the concurrent validity of a novel surface electromyography system (MR EMG, Dunedin, New Zealand) relative to a laboratory-grade reference system (Trigno™ Wireless System, Delsys, Inc., Natick, MA, USA). Participants attended the laboratory for two visits, approximately one week apart. During the first visit, anthropometric data were collected, followed by a dynamic warm-up consisting of 10 quadriceps pulls, 10 bodyweight squats, and 10 walking lunges per leg. Next, a prediction maximal strength test on a plate-loaded leg extension machine (LE08, GMWD Fitness, Denver, CO, USA) was conducted to determine a predicted 1RM.

**Figure 1:**
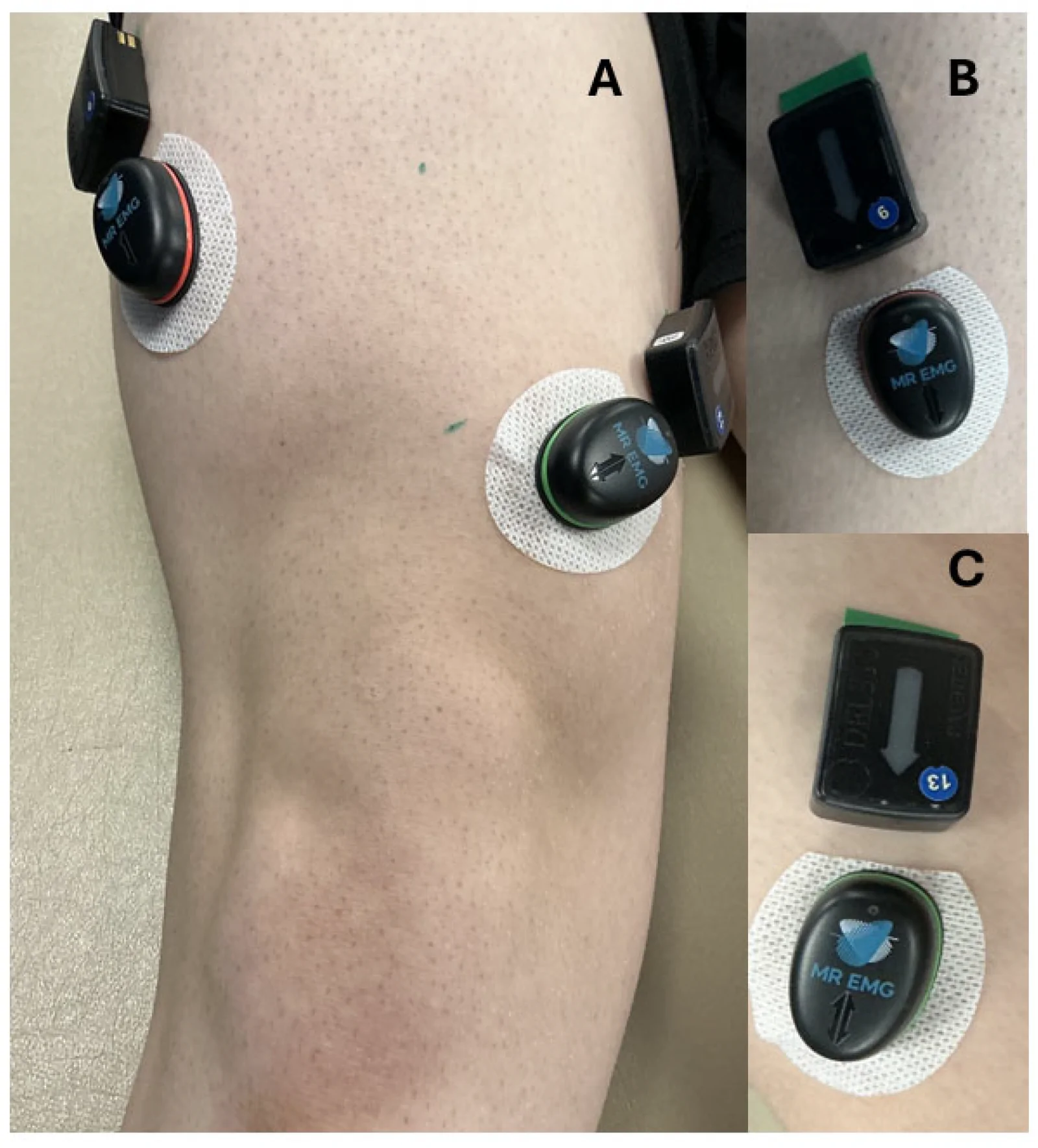
Representation of MR EMG and Delsys sensor placement over the anterior thigh, with the combined view shown in (**A**), the vastus lateralis in (**B**), and the vastus medialis obliquus in (**C**), illustrating relative electrode positioning.

During the second visit, participants completed the same dynamic warm-up prior to electrode placement. Electrodes were positioned on the right leg over the VL and VMO per Lotfi, Moghadam [24]. After electrode placement sites were identified, the skin was shaved and prepared. Per the manufacturers’ recommendations, MR EMG electrode sites were prepared with moisturizer, and Delsys Trigno electrode sites were prepared with an alcohol pad. The MR EMG electrode was placed directly over the anatomical landmark for each muscle. The Delsys Trigno electrode was then placed 1–1.5 cm superior along the muscle fiber axis relative to the MR EMG electrode (Figure 1). The VMO electrode was positioned 4 cm superior to the patella and 3 cm medial, with a 55° medial orientation. The VL electrode was positioned 10 cm superior to the patella and 7 cm lateral, with a 15° medial orientation.

Following electrode placement, participants performed two 5-s maximal voluntary isometric contractions (MVICs) for each muscle to establish normalization values. The highest MVIC value for each muscle across the two trials was recorded. Participants then completed four dynamic repetitions at 30%, 60%, and 90% of their predicted 1RM on the plate-loaded leg extension machine, with EMG amplitude recorded simultaneously by both systems across all conditions.

### 2.3. Maximal Strength Testing

To determine each participant’s relative load, a repetition-to-failure strength test was conducted under the supervision of a certified strength and conditioning specialist. After the dynamic warm-up, participants performed an as-many-repetitions-as-possible (AMRAP) test with loads adjusted across successive sets until a set of 10 or fewer repetitions to failure was achieved within four attempts. Rest intervals were 2 min between sets and 3 min before the final test set. All repetitions were performed through a full range of motion permitted by the leg extension machine at a pace governed by a 40 beats·min^−1^ metronome. Each beat corresponded to one movement phase, such that participants performed the concentric phase on one beat and the eccentric phase on the following beat, yielding a phase duration of approximately 1.5 s and a total repetition time of approximately 3 s. Failure was defined as the inability to maintain the metronome cadence or to complete a repetition through the full range of motion. This protocol was designed to avoid direct 1RM attempts, reducing the risk of quadriceps fatigue and potential tendon or soft-tissue strain associated with maximal single-limb knee extension. The predicted 1RM was estimated from the test set using the Brzycki formula [25]: predicted 1RM = weight lifted/(1.0278 − 0.0278 × repetitions completed).

### 2.4. Maximal Voluntary Isometric Contraction

**Figure 2:**
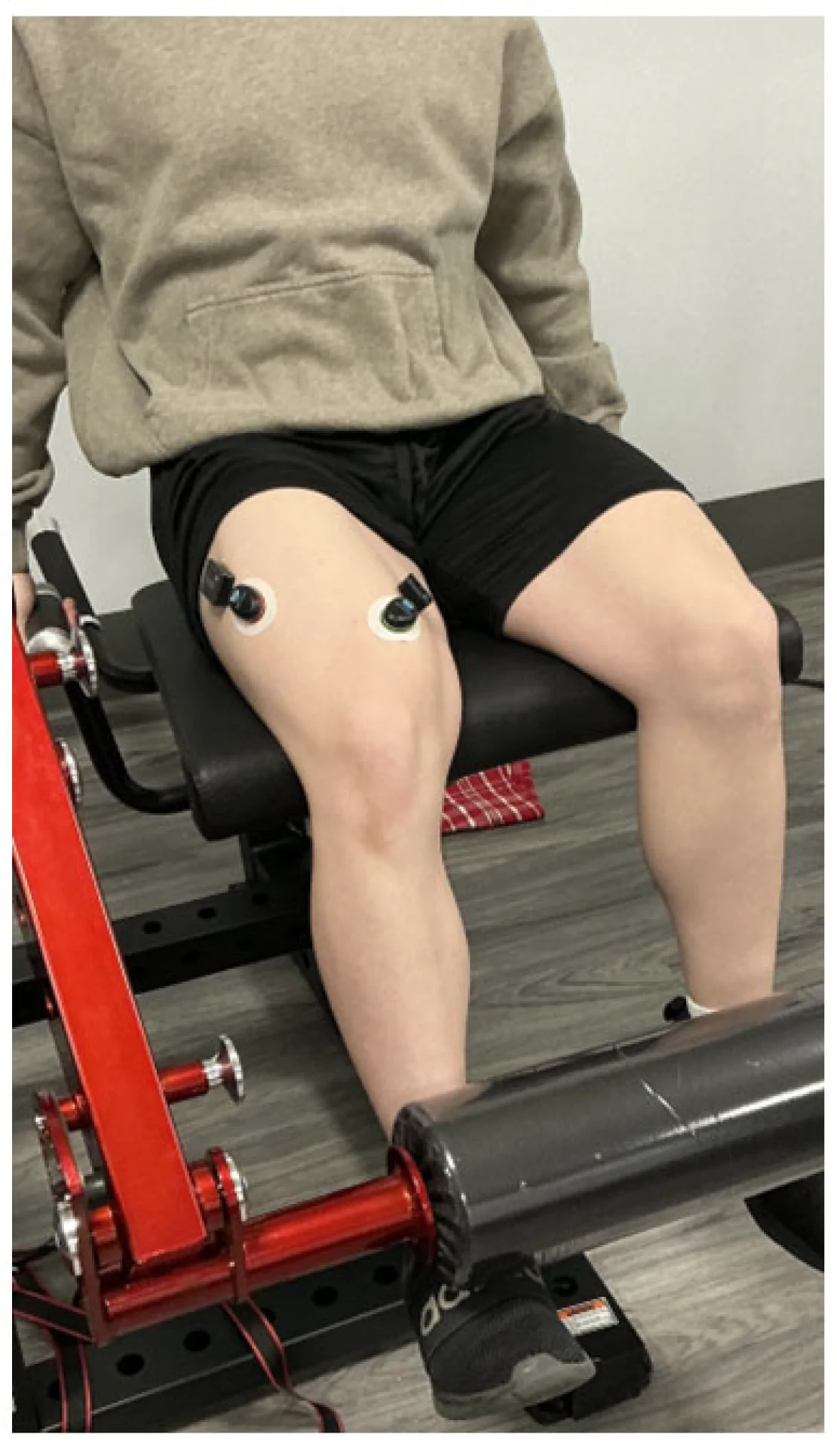
Representative participant positioning during isometric leg extension testing at 60° of knee flexion with concurrent MR EMG and Delsys sensor placement.

MVIC data were obtained to normalize EMG signals [26]. Participants were seated in the leg extension machine with the hip stabilized and the knee positioned at approximately 60–65° of flexion (0° = full extension, Figure 2), a joint angle selected based on evidence of greater peak knee-extensor torque and stable EMG characteristics at mid-range positions [27]. The lower leg was secured against the machine’s resistance pad just above the ankle, and the machine was secured to fixed anchor points with heavy-duty ratchet straps prior to testing. Participants performed a 2-s progressive ramp contraction, followed immediately by a 5-s MVIC, with two trials completed and 3 min of rest between attempts. The highest RMS amplitude identified within a 500-ms window around peak activation across the two trials was used as the session-specific normalization value.

### 2.5. Resistance Exercise Comparison Sets

Following MVIC, participants completed four controlled repetitions at 30%, 60%, and 90% 1RM, separated by 2 to 3 min of rest. Loading conditions were performed in a fixed ascending order for all participants. Signals from both EMG systems were recorded simultaneously during each set. Repetition cadence was standardized using a metronome (40 beats·min^−1^) to control contraction velocity, as sEMG amplitude during dynamic tasks is influenced by biomechanical factors, such as muscle length and contraction velocity [5]. The first repetition was excluded from analysis, as initiating from a deep knee-flexion position was not representative of subsequent steady-state repetitions. EMG amplitude from repetitions 2 through 4 was used for all reliability and validity analyses.

### 2.6. Signal Processing and Analysis

#### 2.6.1. MR EMG

**Figure 3:**
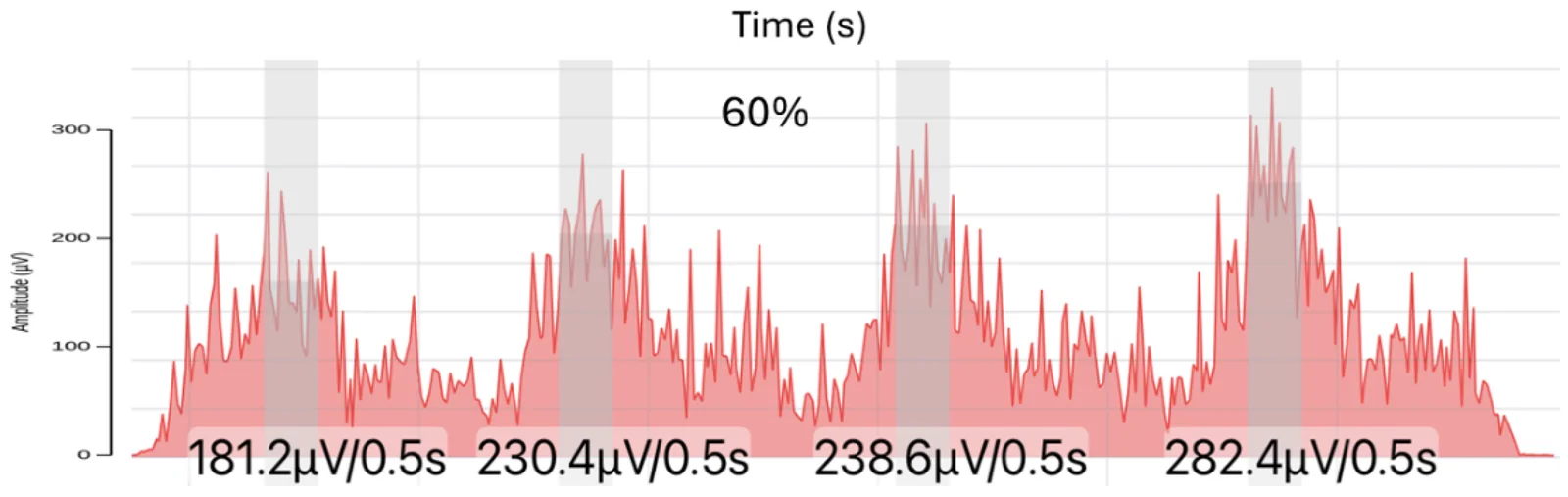
Representative rectified surface electromyography (sEMG) signal recorded from the vastus lateralis during four consecutive repetitions at 60% of one-repetition maximum (1RM) using the MR EMG system. Gray shaded regions denote the 500-ms windows of peak root mean square (RMS) amplitude identified within each repetition and used for analysis. Values represent mean RMS amplitude (µV) within each window.

Prior to data collection, signal quality was confirmed by ensuring that the resting amplitude was below 8 μV, per the manufacturer’s guidelines. For the Delsys Trigno, signal quality was verified visually by confirming a flat resting baseline prior to recording. The MR EMG utilizes disc electrodes; the Delsys Trigno utilizes bar electrodes. Both systems employ bipolar differential detection. The MR EMG system sampled raw EMG signals at 1000 Hz using a 24-bit analog-to-digital converter. The device was configured with a driven right-leg circuit for common-mode noise rejection (a noise-cancellation technique that reduces electrical interference common to both electrodes) and onboard direct-current offset removal to minimize electrode–skin interface drift. Hardware-level filtering was applied during acquisition per manufacturer specifications (Fast Mode high-pass setting 1; Sensor low-pass setting 4). Amplitude estimation was performed using a block RMS computation over a fixed 500-ms interval derived from the filtered signal. For each repetition, a 500-ms window corresponding to the peak phase of the leg extension was manually identified via visual inspection of the rectified signal and selected by the primary investigator using a standardized procedure within the MR EMG application (Figure 3).

#### 2.6.2. Delsys Trigno System

**Figure 4:**
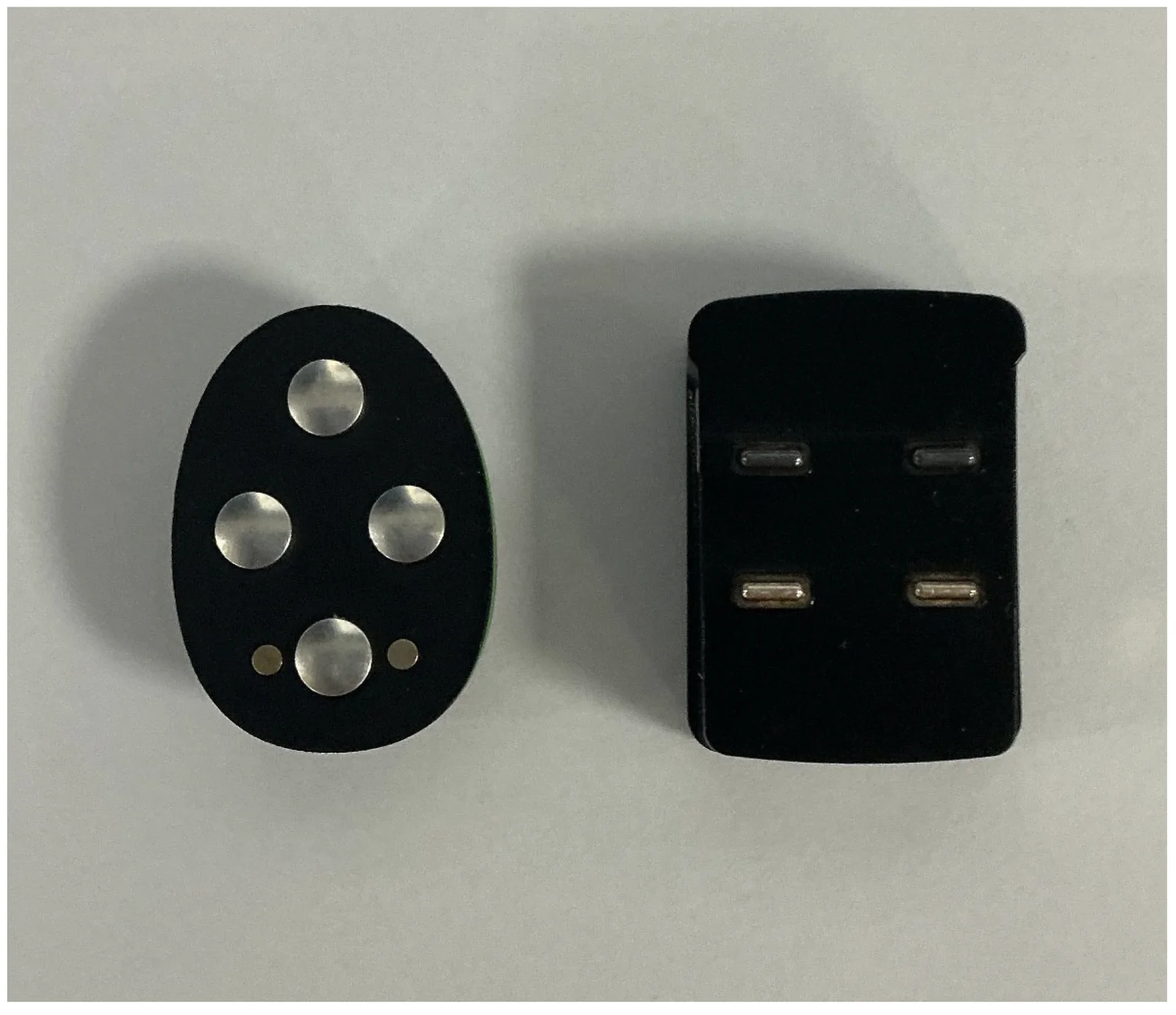
Electrode contact surfaces of the MR EMG (left) and Delsys Trigno (right), illustrating differences in contact geometry and interelectrode configuration between the two devices.

Raw EMG signals from the Delsys Trigno system were sampled at 2148 Hz and imported into EMGworks (Delsys Inc., Natick, MA, USA) for offline processing. The DC offset (a small, constant voltage shift at the electrod–skin interface unrelated to the physiological signal) was removed using the built-in “Remove Offset” function prior to filtering. Signals were band-pass filtered (20–450 Hz, Butterworth), full-wave rectified, and processed using a 500-ms block RMS calculation applied to the peak phase of each repetition. RMS amplitude values from both systems were normalized to MVIC prior to statistical analysis. Although Delsys signals were processed offline in EMGworks and MR EMG amplitude values were computed in real time within the MR EMG application, both approaches applied an equivalent 500-ms block RMS estimation method to the same phase of each repetition. Technical specifications and inter-electrode geometry for both systems are reported in Table 1 and Figure 4.

### 2.7. Synchronization Procedure

Both EMG systems were synchronized using a manual start procedure. The Delsys Trigno system was programmed with a 15-s countdown timer, and the MR EMG recording was initiated when the countdown reached 2 s. Precise sub-second synchronization between systems was not required for a valid comparison, as both systems independently captured the same repetition and the same peak phase of muscle activation within that repetition; the comparison of interest was between-device agreement on a shared biomechanical event, not exact timing alignment.

### 2.8. Statistical Analysis

Reliability and concurrent validity analyses were conducted on raw RMS EMG amplitude (μV) rather than %MVIC-normalized values, as normalization introduces variability tied to each device’s independent MVIC reference, which could confound device-level agreement. Within-session reliability was assessed across repetitions 2 through 4 within each loading condition, separately for each device and muscle, reflecting measurement consistency across repeated efforts rather than test–retest reliability across sessions; inter-repetition physiological variability was treated as a component of, rather than a threat to, this estimate. Descriptive statistics, including means and standard deviations, were calculated for RMS EMG amplitude and MVIC normalization values for each device, muscle, and loading condition. Reliability was quantified using the intraclass correlation coefficient (ICC), coefficient of variation (CV%), and standard error of measurement (SEM). ICC estimates and their 95% confidence intervals were calculated using a two-way mixed-effects model, with absolute agreement based on single measurements across three repetitions (ICC_3,1_) [23]. ICC values were interpreted according to established benchmarks [23]. CV% was calculated as the standard deviation of the three repetitions divided by the mean, multiplied by 100, with 95% confidence intervals estimated using a bootstrap procedure. Two outlying CV values from the same participant and trial were identified and removed prior to analysis, exceeding a *z*-score threshold of 3.0 (z = 3.3 and 4.0). SEM was calculated as the square root of the mean square error derived from a two-way repeated measures ANOVA (SEM = √MSE). Raw RMS amplitude agreement between devices during the isometric MVIC, computed separately for the VL and VMO, is reported in Table 2. All statistical analyses were performed using SPSS (version 30.0; IBM Corp., Armonk, NY, USA).

Concurrent validity between devices was assessed separately for each muscle and loading condition using ICC, Bland–Altman analysis, and Pearson correlations. ICC estimates used a two-way mixed-effects model, with consistency based on single measures (ICC_3,1_), selected a priori because systematic bias between devices was anticipated due to differences in electrode placement and hardware [23]. Given the excellent within-session reliability demonstrated for both systems, the mean RMS EMG amplitude across repetitions 2 through 4 was considered a stable representation of muscle activation for each muscle and loading condition. Bland-Altman analysis was performed by plotting the difference in mean RMS EMG amplitude between devices against the grand mean, separately for each muscle and loading condition. Mean bias and 95% limits of agreement (LOA = mean bias ± 1.96 SD) were reported for each muscle-by-load combination. Proportional bias was assessed by regressing the between-device difference on the grand mean; the slope (*β*) and its 95% confidence interval were reported, with a slope significantly different from zero, indicating that disagreement varied systematically with signal amplitude. Pearson correlation coefficients and their 95% confidence intervals were calculated as a supplementary measure of relative validity [12], with strength interpreted as weak (*r* < 0.30), moderate (0.30–0.59), large (0.60–0.79), or very large (*r* ≥ 0.80) [28].

## 3. Results

### 3.1. Between-Device Agreement During Maximal Voluntary Isometric Contraction

Raw RMS EMG amplitude recorded during the MVIC was compared between devices for each muscle to assess agreement under static, non-loaded conditions. Between-device agreement was good for the VMO (ICC = 0.85 [95% CI: 0.72–0.93]; r = 0.86 [95% CI: 0.72–0.93]) but poor to moderate for the VL (ICC = 0.43 [95% CI: 0.10–0.68]; r = 0.46 [95% CI: 0.13–0.70]) (Table 2).

### 3.2. Within-Session Reliability

#### 3.2.1. Vastus Lateralis

Mean RMS EMG amplitude and %MVIC values for the VL across all loading conditions are presented in Table 3. Within-session reliability was excellent for both devices across all three loading conditions, with ICC values of 0.95 [95% CI: 0.92–0.97], 0.97 [95% CI: 0.94–0.98], and 0.95 [95% CI: 0.91–0.97] for the MR EMG at 30%, 60%, and 90% 1RM, and 0.95 [95% CI: 0.91–0.97], 0.96 [95% CI: 0.92–0.98], and 0.96 [95% CI: 0.92–0.98] for the Delsys. CV% ranged from 7.8% to 9.8% for the MR EMG and from 8.1% to 10.2% for the Delsys across all three conditions. SEM values ranged from 28.6 to 43.3 μV for the MR EMG and from 21.4 to 29.4 μV for the Delsys across loading conditions.

#### 3.2.2. Vastus Medialis Obliquus

Mean RMS EMG amplitude and %MVIC values for the VMO across all loading conditions are presented in Table 3. Within-session reliability was excellent for both devices across all three loading conditions, with ICC values of 0.93 [95% CI: 0.88–0.96], 0.95 [95% CI: 0.91–0.98], and 0.92 [95% CI: 0.87–0.96] for the MR EMG at 30%, 60%, and 90% 1RM, and 0.94 [95% CI: 0.89–0.97], 0.96 [95% CI: 0.88–0.98], and 0.94 [95% CI: 0.87–0.97] for the Delsys. CV% ranged from 8.6% to 9.9% for the MR EMG and from 10.1% to 11.3% for the Delsys across all three conditions, following the removal of two statistical outliers from the same participant and trial at 30% 1RM. SEM values ranged from 43.6 to 69.6 μV for the MR EMG and from 30.0 to 48.0 μV for the Delsys across loading conditions.

### 3.3. Between-Device Consistency

Concurrent validity statistics for both the VL and VMO across all loading conditions are presented in Table 4.

#### 3.3.1. Vastus Lateralis

**Figure 5:**
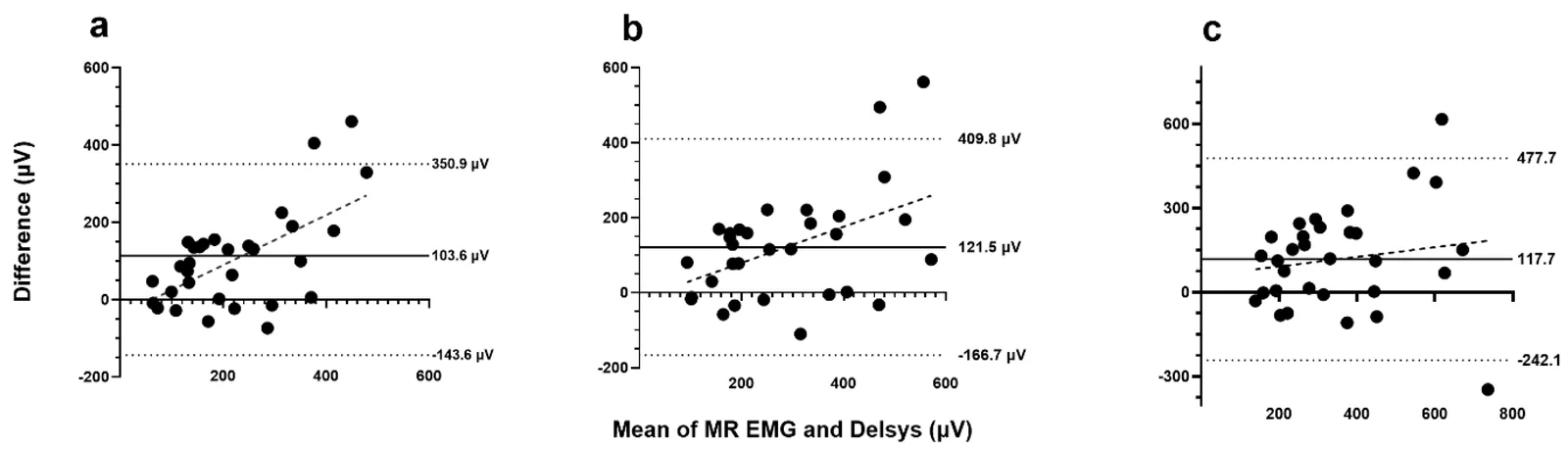
Bland–Altman plots for between-device agreement of vastus lateralis root mean square EMG amplitude at (**a**) 30%, (**b**) 60%, and (**c**) 90% 1RM. The solid horizontal line represents the mean bias, and the dotted lines represent the 95% limits of agreement. The dashed line represents the line of best fit for the difference scores. Note that axis scales differ in (**c**) to accommodate the wider range of values at 90% 1RM. μV = microvolts.

At 30% 1RM, between-device concurrent validity for the VL was moderate (ICC = 0.55 [95% CI: 0.25–0.75]), with a large Pearson correlation (r = 0.63 [95% CI: 0.36–0.81]). The mean bias was 103.6 μV [95% CI: 57.3–149.9], with 95% limits of agreement that ranged from −143.6 to 350.9 μV (Figure 5a), and a regression slope of 0.64 [95% CI: 0.32–0.97]. At 60% 1RM, concurrent validity remained moderate (ICC = 0.57 [95% CI: 0.28–0.77]), with a large Pearson correlation (*r* = 0.62 [95% CI: 0.34–0.81]). The mean bias was 121.5 μV [95% CI: 67.6–175.5], with 95% limits of agreement that ranged from −166.7 to 409.8 μV (Figure 5b), and a regression slope of 0.48 [95% CI: 0.13–0.83]. At 90% 1RM, concurrent validity was moderate (ICC = 0.53 [95% CI: 0.23–0.74]), with a moderate Pearson correlation (r = 0.54 [95% CI: 0.23–0.75]). The mean bias was 117.7 μV [95% CI: 50.4–185.1], with 95% limits of agreement that ranged from −242.1 to 477.7 μV (Figure 5c), and a regression slope of 0.17 [95% CI: −0.24–0.58].

#### 3.3.2. Vastus Medialis Obliquus

**Figure 6:**
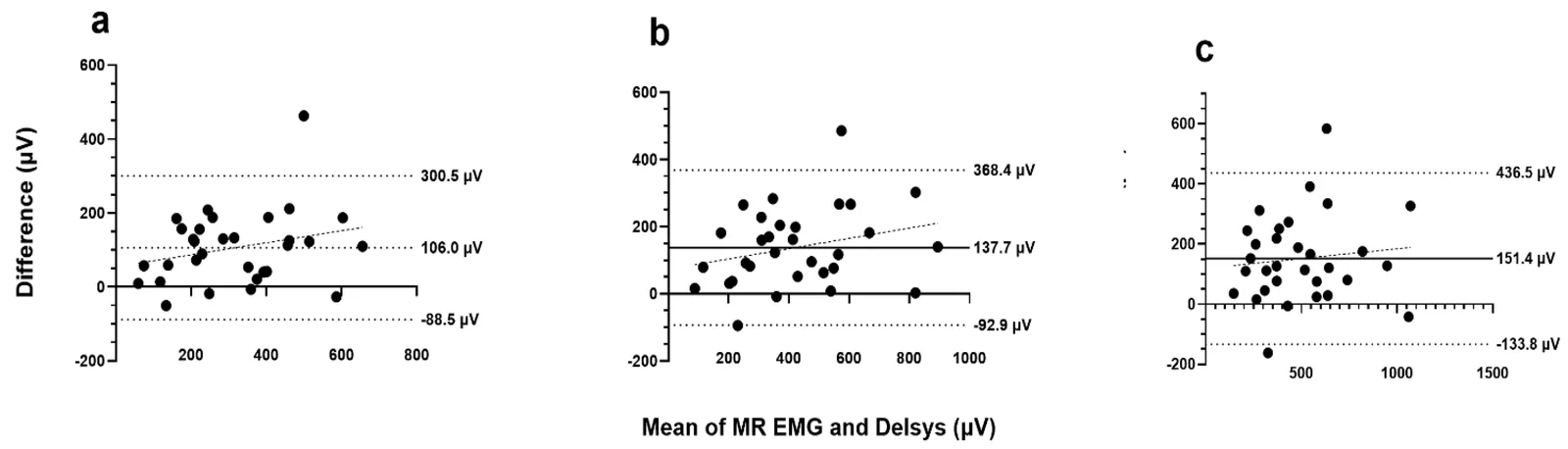
Bland–Altman plots for between-device agreement of vastus medialis obliquus root mean square EMG amplitude at (**a**) 30%, (**b**) 60%, and (**c**) 90% 1RM. The solid horizontal line represents the mean bias, and the dotted lines represent the 95% limits of agreement. The dashed line represents the line of best fit for the difference scores. Note that axis scales differ in (**c**) to accommodate the wider range of values at 90% 1RM. μV = microvolts.

At 30% 1RM, between-device concurrent validity for the VMO was good (ICC = 0.82 [95% CI: 0.67–0.91]) with a large Pearson correlation (*r* = 0.83 [95% CI: 0.68–0.91]). The mean bias was 106.0 μV [95% CI: 69.5–142.3], with 95% limits of agreement that ranged from −88.5 to 300.5 μV (Figure 6a) and a regression slope of 0.16 [95% CI: −0.06–0.38]. At 60% 1RM, concurrent validity remained good (ICC = 0.85 [95% CI: 0.71–0.92]), with a large Pearson correlation (*r* = 0.86 [95% CI: 0.72–0.92]). The mean bias was 137.7 μV [95% CI: 94.5–180.8], with 95% limits of agreement that ranged from −92.8 to 368.4 μV (Figure 6b) and a regression slope of 0.15 [95% CI: −0.05–0.36]. At 90% 1RM, concurrent validity remained good (ICC = 0.84 [95% CI: 0.69–0.91]), with a large Pearson correlation (r = 0.84 [95% CI: 0.69–0.92]). The mean bias was 151.4 μV [95% CI: 97.9–204.7], with 95% limits of agreement that ranged from −133.8 to 436.5 μV (Figure 6c) and a regression slope of 0.06 [95% CI: −0.16–0.29].

## 4. Discussion

Both devices demonstrated excellent within-session reliability across all loading conditions and both muscles, while between-device concurrent validity differed by muscle, with good VMO agreement and moderate VL agreement. The first hypothesis was supported, as within-session reliability ICC values consistently exceeded the good-to-excellent threshold established by Koo and Li [23]. The second hypothesis was partially supported. Concurrent validity for the VMO met the a priori threshold at all three loads, with good ICC values and large Pearson correlations observed across loads. Concurrent validity for the VL, however, did not meet the threshold at any loading condition, with moderate ICC values and systematic overestimation of RMS EMG amplitude by the MR EMG relative to the Delsys Trigno across all three loads. These findings suggest that the performance of the MR EMG is muscle-dependent, with anatomical and methodological factors specific to each recording site influencing the degree of between-device agreement.

This study compared MR EMG and Delsys as each system is actually used, including how each device processes its own signal: MR EMG computes amplitude in real time on its onboard software, while Delsys signals are processed offline in EMGworks. Practitioners use both systems this way, as complete, manufacturer-configured tools rather than bare sensors, so this comparison reflects real-world use rather than an artificial setup where both devices are forced through identical filtering. Some of the agreement differences reported here may come from this processing difference, not just the hardware or electrodes, a point we return to in the Limitations.

Within-session reliability was excellent for both devices across all three loading conditions, consistent with the thresholds reported by Koo and Li [23]. CV% values remained below 12% for both devices across all loading conditions, consistent with the 8–16% range reported for RMS-based amplitude measures during dynamic tasks [29,30]. SEM values should be interpreted in the context of each device’s mean amplitude, as the larger SEM values for the MR EMG partly reflect its higher absolute RMS values rather than inferior measurement precision [31].

The MR EMG demonstrated moderate concurrent validity relative to the Delsys Trigno for the VL across all three loading conditions. Systematic bias was present at all loads, as the mean bias confidence intervals did not contain zero, indicating consistent overestimation of RMS EMG amplitude by the MR EMG relative to the Delsys Trigno. Proportional bias was present at 30% and 60% 1RM, as the regression slope confidence intervals did not contain zero at these loads, with between-device differences increasing as a function of signal magnitude. At 90% 1RM, however, the regression slope confidence interval crossed zero, suggesting that proportional bias was not present at the highest loading condition.

The moderate concurrent validity observed for the VL across loading conditions is likely attributable to anatomical and methodological factors specific to this muscle. Despite the MR EMG and Delsys Trigno electrodes being placed in close proximity on the same muscle belly, the two systems differ in electrode contact geometry and interelectrode distance, and each was normalized to its own MVIC reference value. Even small differences in electrode placement and configuration over the VL have been shown to produce meaningfully different amplitude estimates, with normalization alone insufficient to fully resolve these discrepancies, likely because each device captured a slightly different portion of the muscle [32,33,34]. Given the 15° orientation of the VL electrodes along the muscle fiber pennation angle, differences in contact geometry likely caused each system to sample slightly different regions of this large, architecturally complex muscle. Bawa and Banitsas [13] reported good to excellent inter-system agreement on the VL (ICC 0.74 to 0.92) using a tightly controlled parallel co-placement, which suggests that stronger correspondence is achievable when placement conditions are consistent over this muscle. Molina-Molina, Ruiz-Malagón [12] and Lynn, Watkins [11] similarly reported strong VL validity using dynamometer-controlled isokinetic contractions, which constrain joint velocity, range of motion, and torque output, substantially reducing between-repetition movement variability. Although the present study employed a metronome to standardize repetition tempo, this controls only cadence and does not eliminate the between-repetition variability in joint kinematics and muscle recruitment inherent to free dynamic exercise, which likely contributed to the more moderate VL agreement observed here. Beyond electrode placement, the architectural complexity of the VL itself may further account for these findings. High-density EMG studies have demonstrated that VL activation is spatially non-uniform, with region-specific differences in amplitude and distribution that shift with contraction intensity, reflecting heterogeneous motor unit recruitment [35,36]. This heterogeneity may increase sensitivity to electrode placement and contribute to the reduced between-device consistency observed here.

Between-device agreement for the VL was also poor to moderate during the isometric MVIC (Table 2), a static condition with no motion artifact, contraction velocity, or change in muscle length. Disagreement was therefore present even without movement, so motion-related factors alone cannot account for the moderate VL agreement seen during dynamic loading (Table 4). Electrode contact geometry, interelectrode distance, and the architectural complexity of the VL itself likely play a larger role than dynamic movement artifact. The VMO showed good agreement in both static and dynamic conditions (Table 2 and Table 4), consistent with a more discrete, superficial muscle belly that is less sensitive to placement regardless of contraction type.

The good concurrent validity observed for the VMO across all three loading conditions is likely attributable to the muscle’s anatomical characteristics. The VMO presents a more discrete and superficial muscle belly, and with electrodes oriented at 55°, its oblique fiber arrangement was more consistently captured by both devices, reducing the sensitivity to small differences in electrode placement and contact geometry that were observed for the VL [32,33,34]. High-density EMG studies confirm the VMO as a functionally distinct, anatomically discrete region with a contained activation volume that differs from the adjacent VM longus [37], reducing spatial non-uniformity relative to the architecturally complex VL and making amplitude estimates less susceptible to placement differences. The absence of proportional bias across all three loading conditions further supports this interpretation, as between-device differences did not increase with signal magnitude, suggesting that the more consistent electrode capture of the VMO attenuated the amplitude-dependent error observed in the VL. Systematic bias remained, however, indicating that device-level differences in electrode contact geometry and signal processing contributed to a consistent amplitude offset regardless of the muscle.

EMG amplitudes approaching or exceeding MVIC values are consistent with the broader normalization literature, as conventional MVIC procedures have been shown to underestimate true maximal activation during dynamic tasks [38,39,40]. The absolute RMS amplitude and %MVIC values observed in the present study also are consistent with those reported for the VL and VM during knee extension exercise across a range of loading conditions [41,42,43], suggesting that the amplitude ranges fall within expected physiological bounds for these muscles. Although a dynamic normalization approach may have been more appropriate for the present protocol, safety considerations specific to maximal isolated knee extension contractions, including the risk of patellar tendon and joint injury at high loads, precluded their use as a normalization reference.

Several limitations of the present study should be acknowledged. First, the MR EMG and Delsys Trigno electrodes were arranged linearly along the muscle fiber axis rather than placed adjacent to each other at a common recording site, as the VMO’s small cross-sectional area did not permit positioning two electrode systems side by side on the same muscle belly. This linear configuration meant each device sampled a different position along the muscle belly, and studies that found a stronger between-device agreement on the VL have generally placed devices adjacent to one another at a single marked location. Second, the two devices differ inherently in electrode contact geometry and interelectrode distance, which contributed to the systematic amplitude differences observed across muscles and loading conditions, independent of placement. In addition, the MR EMG’s lower high-pass cutoff (1 Hz vs. 20 Hz for Delsys) may have allowed low-frequency movement-related artifacts to contribute to the systematic amplitude overestimation observed during dynamic exercise. The two systems also processed signals differently: MR EMG in real time on its onboard software, Delsys offline in EMGworks. The present findings reflect agreement between complete measurement systems, not electrode performance alone, so some of the observed differences may come from this processing difference rather than the electrodes themselves. Each device’s electrode is itself part of the analog signal path, not just a contact point: the electrode’s physical shape and geometry determine which signal it picks up, before any electronics are involved. The electrode also forms a combined circuit with that device’s amplifier: the voltage that actually reaches the amplifier depends on both the electrode’s properties and the amplifier’s input characteristics together, and this combination differs by manufacturer. Because of this, the electrode cannot be swapped or equalized independently of the processing that follows it. Matching the filtering, sampling rate, or RMS windowing afterward would not fix these earlier differences. A truly identical comparison would require access to each device’s signal before this point, which neither manufacturer allows. For this reason, the present study compares the two systems as complete measurement units, the way each is actually used. Digital-level harmonization was also attempted directly and found unworkable: MR EMG’s signal is rectified onboard prior to export (Figure 3), so applying RMS analysis to it a second time distorts rather than reproduces a valid raw-to-RMS pipeline comparable to Delsys’s. Third, resistance training background was characterized only by years of training experience. The specific type of training performed (e.g., general fitness, sport-specific, powerlifting) was not assessed, and training modality may influence movement execution and muscle activation patterns during the leg extension exercise. Fourth, our findings are specific to the leg extension exercise and may not generalize to other movements without further investigation, as different kinematic and loading demands may produce different agreement patterns. Fifth, the metronome controlled repetition cadence only, not joint kinematics, torque output, or motor unit recruitment across repetitions. Finally, two data points from the same participant and trial were removed as statistical outliers from the within-session reliability analysis at 30% 1RM for the VMO, likely reflecting variability in effort at lower loading intensities. When the two outlying values were retained, the VMO’s reliability classification remained unchanged. Future studies should consider a convergent validity design in which the criterion electrode is repositioned to the exact MR EMG recording site for a follow-up trial, though fatigue and electrode-repositioning demands present practical challenges that should be considered in the study design. Future protocols also should consider submaximal dynamic normalization alternatives to the isometric MVIC procedure, as dynamic reference contractions may better approximate the activation demands of the exercises being assessed. MR EMG concurrent validity should also be evaluated under isokinetic dynamometer-controlled conditions, which standardize the mechanical demands of contraction, though researchers should weigh this against the reduced ecological validity of dynamometer-based protocols relative to free dynamic exercise.

The MR EMG demonstrated excellent within-session reliability for both muscles, supporting its use as a reliable tool for monitoring muscle activation in resistance training. Its wireless design and iPad-based software make it accessible to practitioners outside the laboratory. Concurrent validity was muscle-dependent, with good agreement for the VMO and moderate agreement for the VL. Practitioners can have reasonable confidence in VMO amplitude estimates across loading intensities, while VL amplitude estimates should be interpreted in the context of the placement constraints and architectural complexity specific to this muscle. Overall, the MR EMG demonstrated excellent reliability and moderate-to-good agreement with a laboratory-grade sEMG system, supporting its use for monitoring relative changes in muscle activation during dynamic resistance exercise.

## Figures and Tables

**Table 1:** Technical specifications of the MR EMG and Delsys Trigno surface electromyography systems.

Parameter	MR EMG	Delsys
Dimensions (mm)	34 × 24 × 10	27 × 37 × 13
Interelectrode distance (mm)	8	10
Weight (g)	10	14
Channels	4	16
Bandwidth (Hz)	1–70	20–450
Gain	×3	300 (V/V)
Sampling rate (Hz)	1000	2148
Common mode rejection ratio (dB)	120	>80
Operating voltage (mv)	+/− 800	+/− 5000
Contact electrode	99.9 silver	99.9 silver
Output mode	RMS; raw output available	raw EMG

Note. mm = millimeters; g = grams; Hz = hertz; V/V = volts per volt; mV = millivolts; dB = decibels.

**Table 2:** Raw root mean square (RMS) amplitude and between-device agreement during maximal voluntary isometric contraction (MVIC) for the vastus lateralis (VL) and vastus medialis obliquus (VMO).

Muscle	Device	MVIC RMS Mean ± SD (μV)	ICC [95% CI]	r [95% CI]
VL	MR EMG	325.8 ± 179.1		
VL	Delsys	229.1 ± 125.0	0.43 [0.10–0.68]	0.46 [0.13–0.70]
VMO	MR EMG	477.0 ± 249.9		
VMO	Delsys	372.2 ± 229.8	0.85 [0.72–0.93]	0.86 [0.72–0.93]

Note. ICC = intraclass correlation coefficient (consistency); r = Pearson correlation coefficient; CI = confidence interval; μV = microvolts.

**Table 3:** Within-session reliability of EMG amplitude across load conditions for the vastus lateralis (VL) and vastus medialis obliquus (VMO).

Muscle	Load (%1RM)	Device	Mean ± SD (μV)	MVIC ± SD (%)	CV% [95% CI]	ICC [95% CI]	SEM μV
VL	30	MR EMG	273.8 ± 163.2	84.8 ± 21.6	9.8% [7.4–12.2]	0.95 [0.92–0.97]	33.7
		Delsys	170.1 ± 94.0	75.9 ± 18.1	8.1% [5.8–10.4]	0.95 [0.91–0.97]	21.4
	60	MR EMG	348.2 ± 187.3	110.3 ± 23.2	7.8% [6.0–9.6]	0.97 [0.94–0.98]	28.6
		Delsys	226.7 ± 125.6	100.9 ± 19.6	8.5% [7.1–9.9]	0.96 [0.92–0.98]	22.8
	90	MR EMG	409.4 ± 202.8	133.8 ± 32.8	9.5% [7.5–11.5]	0.95 [0.91–0.97]	43.3
		Delsys	291.6 ± 177.7	131.5 ± 32.2	10.2% [8.2–12.2]	0.96 [0.92–0.98]	29.4
VMO	30	MR EMG	369.5 ± 179.6	84.0 ± 32.6	9.6% [7.9–11.2]	0.93 [0.88–0.96]	45.8
		Delsys	263.5 ± 154.6	76.9 ± 31.1	10.9% [7.8–14.0]	0.94 [0.89–0.97]	39.5
	60	MR EMG	489.3 ± 228.4	110.8 ± 30.8	8.6% [6.8–10.5]	0.95 [0.91–0.98]	43.6
		Delsys	351.7 ± 198.0	102.7 ± 35.1	10.1% [8.6–11.6]	0.96 [0.88–0.98]	30.0
	90	MR EMG	571.2 ± 262.1	138.2 ± 41.8	9.9% [7.9–11.9]	0.92 [0.87–0.96]	69.6
		Delsys	419.9 ± 246.9	124.8 ± 33.3	11.3% [8.8–13.9]	0.94 [0.87–0.97]	48.0

Note. RM = repetition maximum; SD = standard deviation; MVIC = maximal voluntary isometric contraction; CV = coefficient of variation; CI = confidence interval; ICC = intraclass correlation coefficient (absolute agreement); SEM = standard error of measurement; µV = microvolts.

**Table 4:** Between-device concurrent validity of EMG amplitude across load conditions for the vastus lateralis (VL) and vastus medialis obliquus (VMO).

Muscle	Load (%1RM)	ICC [95% CI]	r [95% CI]	Bias (μV) [95% CI]	LOA (μV)	β [95% CI]
VL	30	0.55 [0.25–0.75]	0.63 [0.36–0.81]	103.6 [57.3–149.9]	−143.6–350.9	0.64 [0.32–0.97]
	60	0.57 [0.28–0.77]	0.62 [0.34–0.81]	121.5 [67.6–175.5]	−166.7–409.8	0.48 [0.13–0.83]
	90	0.53 [0.23–0.74]	0.54 [0.23–0.75]	117.7 [50.4–185.1]	−242.1–477.7	0.17 [−0.24–0.58]
VMO	30	0.82 [0.67–0.91]	0.83 [0.68–0.91]	106.0 [69.5–142.3]	−88.5–300.5	0.16 [−0.06–0.38]
	60	0.85 [0.71–0.92]	0.86 [0.72–0.92]	137.7 [94.5–180.8]	−92.8–368.4	0.15 [−0.05–0.36]
	90	0.84 [0.69–0.91]	0.84 [0.69–0.92]	151.4 [97.9–204.7]	−133.8–436.5	0.06 [−0.16–0.29]

Note. RM = repetition maximum; ICC = intraclass correlation coefficient (consistency); r = Pearson correlation coefficient; CI = confidence interval; µV = microvolts; β = slope of the regression line from Bland–Altman analysis; LOA = limits of agreement.

## Data Availability

The data that support the findings of this study are available from the corresponding author upon reasonable request.

## References

[1] Petrofsky J.S., Glaser R.M., Phillips C.A., Lind A.R., Williams C. (1982). Evaluation of amplitude and frequency components of the surface EMG as an index of muscle fatigue. Ergonomics.

[2] Scano A., Lanzani V., Brambilla C. (2024). How Recent Findings in Electromyographic Analysis and Synergistic Control Can Impact on New Directions for Muscle Synergy Assessment in Sports. Appl. Sci..

[3] Clancy E.A., Morin E.L., Hajian G., Merletti R. (2023). Tutorial. Surface electromyogram (sEMG) amplitude estimation: Best practices. J. Electromyogr. Kinesiol..

[4] Farina D., Merletti R., Enoka R.M. (2025). The extraction of neural strategies from the surface EMG: 2004–2024. J. Appl. Physiol..

[5] Vigotsky A.D., Halperin I., Lehman G.J., Trajano G.S., Vieira T.M. (2018). Interpreting Signal Amplitudes in Surface Electromyography Studies in Sport and Rehabilitation Sciences. Front. Phys..

[6] Merletti R., Cerone G.L. (2020). Tutorial. Surface EMG detection, conditioning and pre-processing: Best practices. J. Electromyogr. Kinesiol..

[7] Fortune B.C., Pretty C.G., Chatfield L.T., McKenzie L.R., Hayes M.P. (2019). Low-cost active electromyography. HardwareX.

[8] Gohel V., Mehendale N. (2020). Review on electromyography signal acquisition and processing. Biophys. Rev..

[9] Gomez-Correa M., Cruz-Ortiz D. (2022). Low-Cost Wearable Band Sensors of Surface Electromyography for Detecting Hand Movements. Sensors.

[10] Jang M.H., Ahn S.J., Lee J.W., Rhee M.-H., Chae D., Kim J., Shin M.J. (2018). Validity and Reliability of the Newly Developed Surface Electromyography Device for Measuring Muscle Activity during Voluntary Isometric Contraction. Comput. Math. Methods Med..

[11] Lynn S.K., Watkins C.M., Wong M.A., Balfany K., Feeney D.F. (2018). Validity and Reliability of Surface Electromyography Measurements from a Wearable Athlete Performance System. J. Sports Sci. Med..

[12] Molina-Molina A., Ruiz-Malagón E.J., Carrillo-Pérez F., Roche-Seruendo L.E., Damas M., Banos O., García-Pinillos F. (2020). Validation of mDurance, A Wearable Surface Electromyography System for Muscle Activity Assessment. Front. Physiol..

[13] Bawa A., Banitsas K. (2022). Design Validation of a Low-Cost EMG Sensor Compared to a Commercial-Based System for Measuring Muscle Activity and Fatigue. Sensors.

[14] Matsuda T., Miyamori T., Fujino Y., Nozu S., Kajiwara Y. (2024). Reliability and validity of muscle activity analysis using wearable electromyographs. J. Phys. Ther. Sci..

[15] Jayaraman C., Mummidisetty C.K., Jayaraman A., Pfleeger K., Jacobson M., Ceruolo M., Sen-Gupta E., Caccese J., Chen D. (2024). Validity and reliability study of a novel surface electromyography sensor using a well-consolidated electromyography system in individuals with cervical spinal cord injury. Spinal Cord.

[16] Tecchio P., Monte A., Zamparo P. (2021). Low-cost electromyography: Validity against a commercial system depends on exercise type and intensity. Eur. J. Transl. Myol..

[17] Prasad S., Paulin M., Cannon R.D., Palla S., Farella M. (2019). Smartphone-assisted monitoring of masticatory muscle activity in freely moving individuals. Clin. Oral Investig..

[18] Francois G., Farella M., Bennani H. (2026). The Effect of Clear Aligners on Awareness of Tooth Clenching. J. Oral Rehabil..

[19] Hu A., Firth F., Bennani H., Iodice G., Farella M. (2026). The effect of clear aligners with posterior occlusal attachments on daytime masticatory muscle activity. Am. J. Orthod. Dentofac. Orthop..

[20] Pittar N., Firth F., Bennani H., Farella M. (2023). The effect of passive clear aligners on masticatory muscle activity in adults with different levels of oral parafunction. J. Oral Rehabil..

[21] Kidwell J.A., Yamamoto T., Hetherton K.J., Truneh N., Bright J.J., Blatney A.E., Goldman P., Neufeld E., Dolezal B.A. (2026). Acute Effects of Thoracic-Spinal Elevation via a Novel Bench Press Pad on sEMG and Barbell Kinetics in Resistance-Trained Males. Int. J. Exerc. Sci..

[22] Bujang M.A., Baharum N. (2017). A simplified guide to determination of sample size requirements for estimating the value of intraclass correlation coefficient: A review. Arch. Orofac. Sci..

[23] Koo T.K., Li M.Y. (2016). A Guideline of Selecting and Reporting Intraclass Correlation Coefficients for Reliability Research. J. Chiropr. Med..

[24] Lotfi H., Moghadam A.N., Shati M. (2021). Electromyography Activity of Vastus Medialis Obliquus and Vastus Lateralis Muscles During Lower Limb Proprioceptive Neuromuscular Facilitation Patterns in Individuals with and without Patellofemoral Pain Syndrome. Phys. Ther. Res..

[25] Brzycki M. (1993). Strength Testing—Predicting a One-Rep Max from Reps-to-Fatigue. J. Phys. Educ. Recreat. Dan..

[26] Burden A. (2010). How should we normalize electromyograms obtained from healthy participants? What we have learned from over 25 years of research. J. Electromyogr. Kinesiol..

[27] de Sousa A.M.M., Cavalcante J.G.T., Bottaro M., Vieira D.C.L., Babault N., Geremia J.M., Corrigan P., Silbernagel K.G., Durigan J.L.Q., Marqueti R.d.C. (2023). The Influence of Hip and Knee Joint Angles on Quadriceps Muscle-Tendon Unit Properties during Maximal Voluntary Isometric Contraction. Int. J. Environ. Res. Public Health.

[28] Hopkins W.G., Marshall S.W., Batterham A.M., Hanin J. (2009). Progressive statistics for studies in sports medicine and exercise science. Med. Sci. Sports Exerc..

[29] Carius D., Kugler P., Kuhwald H.-M., Wollny R. (2015). Absolute and relative intrasession reliability of surface EMG variables for voluntary precise forearm movements. J. Electromyogr. Kinesiol..

[30] Yáñez García J., Rosell D.R., Custodio R.M., Ravelo García A.G., Serna J.R., González Badillo J.J. (2018). Reliability of Mechanical and EMG Variables Assessed During Concentric Bench Press Exercise Against Different Submaximal Loads. Biomed. J. Sci. Tech. Res..

[31] Weir J.P. (2005). Quantifying test-retest reliability using the intraclass correlation coefficient and the SEM. J. Strength Cond. Res..

[32] Beck T.W., Housh T.J., Cramer J.T., Weir J.P. (2008). The effects of electrode placement and innervation zone location on the electromyographic amplitude and mean power frequency versus isometric torque relationships for the vastus lateralis muscle. J. Electromyogr. Kinesiol..

[33] Beck T.W., Housh T.J., Cramer J.T., Weir J.P. (2009). The effects of interelectrode distance over the innervation zone and normalization on the electromyographic amplitude and mean power frequency versus concentric, eccentric, and isometric torque relationships for the vastus lateralis muscle. J. Electromyogr. Kinesiol..

[34] Wong Y.M., Ng G. (2006). Surface electrode placement affects the EMG recordings of the quadriceps muscles. Phys. Ther. Sport.

[35] Pradhan A., Malagon G., Lagacy R., Chester V., Kuruganti U. (2020). Effect of age and sex on strength and spatial electromyography during knee extension. J. Physiol. Anthropol..

[36] Watanabe K., Kouzaki M., Merletti R., Fujibayashi M., Moritani T. (2012). Spatial EMG potential distribution pattern of vastus lateralis muscle during isometric knee extension in young and elderly men. J. Electromyogr. Kinesiol..

[37] Guzman-Venegas R., Valencia O., Cadore E.L., Izquierdo M. (2021). Neuromuscular Compartmentalization of the Vastus Medialis Muscle: Comparison of the Activity of the Vastus Medialis Obliquus and the Vastus Medialis Longus by High Density Electromyography. Int. J. Morphol..

[38] Wang X., Martinez K.B., Golabchi A., Tavakoli M., Rouhani H. (2023). A Dynamic Procedure to Detect Maximum Voluntary Contractions in Low Back. Sensors.

[39] Chuang T.D., Acker S.M. (2019). Comparing functional dynamic normalization methods to maximal voluntary isometric contractions for lower limb EMG from walking, cycling and running. J. Electromyogr. Kinesiol..

[40] Korak J.A., Bruininks B.D., Paquette M.R. (2020). The Influence of Normalization Technique on Between-Muscle Activation during a Back-Squat. Int. J. Exerc. Sci..

[41] Alkner B., Tesch P.A., Berg H.E. (2000). Quadriceps EMG/force relationship in knee extension and leg press. Med. Sci. Sports Exerc..

[42] Stien N., Saeterbakken A.H., Andersen V. (2021). Electromyographic Comparison of Five Lower-Limb Muscles between Single- and Multi-Joint Exercises among Trained Men. J. Sports Sci. Med..

[43] Muyor J.M., Martín-Fuentes I., Rodríguez-Ridao D., Antequera-Vique J.A. (2020). Electromyographic activity in the gluteus medius, gluteus maximus, biceps femoris, vastus lateralis, vastus medialis and rectus femoris during the Monopodal Squat, Forward Lunge and Lateral Step-Up exercises. PLoS ONE.

